# Alpha-Class Glutathione S-Transferases in Wild Turkeys (*Meleagris gallopavo*): Characterization and Role in Resistance to the Carcinogenic Mycotoxin Aflatoxin B_1_

**DOI:** 10.1371/journal.pone.0060662

**Published:** 2013-04-16

**Authors:** Ji Eun Kim, Brett R. Bunderson, Amanda Croasdell, Kent M. Reed, Roger A. Coulombe

**Affiliations:** 1 Department of Animal, Dairy, and Veterinary Sciences and Interdepartmental Graduate Toxicology Program, Utah State University, Logan, Utah, United States of America; 2 Department of Veterinary and Biomedical Sciences, College of Veterinary Medicine, University of Minnesota, St. Paul, Minnesota, United States of America; University of Sydney, United States of America

## Abstract

Domestic turkeys (*Meleagris gallopavo*) are one of the most susceptible animals known to the toxic effects of the mycotoxin aflatoxin B_1_ (AFB_1_), a potent human hepatocarcinogen, and universal maize contaminant. We have demonstrated that such susceptibility is associated with the inability of hepatic glutathione S-transferases (GSTs) to detoxify the reactive electrophilic metabolite *exo*-AFB_1_-8,9-epoxide (AFBO). Unlike their domestic counterparts, wild turkeys, which are relatively AFB_1_-resistant, possess hepatic GST-mediated AFBO conjugating activity_._ Here, we characterized the molecular and functional properties of hepatic alpha-class GSTs (GSTAs) from wild and domestic turkeys to shed light on the differences in resistance between these closely related strains. Six alpha-class GST genes (*GSTA*) amplified from wild turkeys (Eastern and Rio Grande subspecies), heritage breed turkeys (Royal Palm) and modern domestic (Nicholas strain) turkeys were sequenced, and catalytic activities of heterologously-expressed recombinant enzymes determined. Alpha-class identity was affirmed by conserved GST domains and four signature motifs. All *GSTAs* contained single nucleotide polymorphisms (SNPs) in their coding regions: *GSTA1.1* (5 SNPs), *GSTA1.2* (7), *GSTA1.3* (3), *GSTA2* (3), *GSTA3* (1) and *GSTA4* (2). *E. coli*-expressed GSTAs possessed varying activities toward GST substrates 1-chloro-2,4-dinitrobenzene (CDNB), 1,2-dichloro-4-nitrobenzene (DCNB), ethacrynic acid (ECA), cumene hydroperoxide (CHP). As predicted by their relative resistance, livers from domestic turkeys lacked detectable GST-mediated AFBO detoxification activity, whereas those from wild and heritage birds possessed this critical activity, suggesting that intensive breeding and selection resulted in loss of AFB_1_-protective alleles during domestication. Our observation that recombinant tGSTAs detoxify AFBO, whereas their hepatic forms do not, implies that the hepatic forms of these enzymes are down-regulated, silenced, or otherwise modified by one or more mechanisms. These data may inform of possible molecular mechanisms of resistance to AFB_1_, and may also have the benefit of identifying genetic markers which could be used to enhance AFB_1_ resistance in modern domestic strains.

## Introduction

Aflatoxin B_1_ (AFB_1_), produced by *Aspergillus* fungi, is a ubiquitous dietary hepatotoxin and hepatocarcinogen, and a major public health concern worldwide, especially in locales where contaminated commodities, such as corn and peanuts, are consumed as a dietary staple [1], [2]. The annual global burden of human hepatocellular carcinoma caused by AFB_1_ is as high as 155,000 cases, with most occurring in sub-Saharan Africa, Southeast Asia and China [3]. Aflatoxin B_1_ was discovered in the early 1960 s as the principal etiological agent of ‘Turkey X Disease’ responsible for the deaths of poultry throughout Europe as a result of contaminated peanut-based meal [1], [4], [5]. There are considerable species-specific differences with respect to susceptibility to the toxic effects of AFB_1_, and domestic turkeys (*Meleagris gallopavo*) are one of the most susceptible species known. Because of their extreme sensitivity, turkeys have been posited as effective surrogates to study AFB_1_ susceptible human populations [6].

The mycotoxin AFB_1_ is not toxic *per se*, but requires enzymatic “bioactivation” by hepatic cytochromes P450 (P450 s) that catalyze the formation of the electrophilic, carcinogenic and mutagenic intermediate the aflatoxin B_1_-8,9-epoxide (AFBO) [2], [6]. Turkeys bioactivate AFB_1_ primarily by high-efficiency P450 s 1A5 and 3A37 which we have recently cloned, sequenced and functionally characterized [6]–[8]. At pharmacological AFB_1_ concentrations, P450 1A5 is responsible for more than 98% of the bioactivation in turkey liver [6].

In humans and most animals, the major AFB_1_ detoxification route is via conjugation of the AFBO to endogenous glutathione (GSH) catalyzed by the classical detoxification enzymes glutathione S-transferases (EC 2.5.1.18: GSTs). This family of multifunctional proteins is involved in phase II metabolism and detoxification of electrophilic xenobiotics such as anti-cancer drugs, chemical carcinogens and environmental pollutants [9], [10]. While mice efficiently bioactivate AFB1, they are remarkably AFB1-resistant owing to the expression of the A3 subunit (mGSTA3) which has high catalytic activity toward AFBO [11]. GSTA3 knockout mice are exquisitely sensitive to the toxic and genotoxic effects of AFB_1_ compared to the wild type [12] affirming the centrality of GSTs in species susceptibility toward AFB_1_. Current consensus is that efficiency of GST conjugation is a principal “rate-limiting” determinant for AFB1 action in individuals and species regardless of the efficiency of AFB_1_ bioactivation [13]. Studies in our laboratory have consistently demonstrated that GSTs from the livers of domestic turkeys have little or no AFBO affinity activity toward AFB_1,_ a condition postulated as an important mechanism for their extreme sensitivity [2], [14]–[16]. Comparative toxicology studies have shown that wild turkeys are substantially more resistant to AFB_1_ compared to domestic turkeys [17]. Reflecting this relative resistance, livers from wild turkeys, unlike their domestic counterparts, possess functional, AFBO-detoxifying GSTs [18]. It is possible that AFB_1_-protective GST alleles were inadvertently lost through years of intensive breeding, and industry consolidation to produce the modern domestic turkey. Indeed, it has been reported that similar breeding pressures have resulted in a marked loss of rare alleles and genetic diversity of SNPs in commercial breeds of chickens [19].

Domestic turkeys possess six alpha-class GST genes (*tGSTA*s) the homologues to AFB_1_-protective GSTAs in mice and in other species [20], [21]. Unlike the hepatic forms of turkey GSTs, which show no detectable AFBO-trapping ability, all recombinant and heterologously-expressed tGSTAs possessed measurable and comparable AFBO-conjugating activity; further, there is no apparent correlation between individual amino acid residues and AFBO-conjugation activity among the tGSTAs [21]. The purpose of this study was to determine whether there are genotypic and functional differences of hepatic *GSTAs* cloned, and heterologously expressed from two subspecies of wild turkeys, a “heritage” line and, domestic birds that might shed light on molecular evolution of this important disease-protective gene, as well as on the molecular mechanisms of resistance to AFB_1_.

## Methods

### Tissues

Fresh livers were obtained from two subspecies of wild turkey: the Eastern wild (“EW”; *Meleagris gallopavo silvestris*) common to the eastern half of the United States; and the Rio Grande wild (“RGW”; *M.g. intermedia*) native to the central and southern plains states, western Texas, and northeastern Mexico [22]. Livers from Royal Palm (“RP”), a heritage breed developed in the early twentieth century [22], were included as a representative of an early domesticated breed. These livers were flash-frozen in liquid N_2_, shipped overnight in dry ice, and stored at −80°C until use. Livers of modern domestic turkeys (“DT”; *M. gallopavo*; Nicholas strain) were provided by Moroni Feed Cooperative (Moroni, UT). Swiss-Webster mice were obtained from Charles River Laboratories (Wilmington, MA, USA) through Laboratory Animal Research Center, Utah State University and were given a diet containing 0.75% butylated hydroxyanisole (BHA) using corn oil for 14 days. Animals were cared for under institutional approval in an AAALAC-accredited facility. Utah State University’s Animal Use and Care Committee approved all procedures involving animal care, euthanasia and tissue collection.

### RNA Extraction and Gene Amplification

Liver samples were homogenized using a Polytron (Brinkman, Westbury, NY) and mRNA was extracted using Oligotex direct mRNA kit (QIAgen, Valencia, CA). The first strand cDNA was synthesized using SuperScript first-strand synthesis kit for RT-PCR (Invitrogen, Carlsbad, CA). For amplification of the coding region of *GSTA* fragments, PCR was performed using pfuUltra high fidelity DNA polymerase (Stratagene, La Jolla, CA) with gene-specific primers (Table 1). The PCR profile was 2 min at 94°C, 30 s at 94°C, 30 s at annealing temperature 56–60°C (25 cycles), and 90 s at 72°C, followed by a final extension at 72°C for 10 min. Full-length cDNA of each *tGSTA* was subcloned into Zero Blunt PCRII vector (Invitrogen, Carlsbad, CA) and sequenced at Utah State University’s core facility. Sequence data were analyzed using Lasergene SeqManII software (DNASTAR Inc., Madison, WI).

**Table 1:** Summary of primers used to amplify the open reading frame of Alpha-class *GST* fragments from wild and heritage turkey hepatic cDNA.

Gene	Size (bp)	Gene specific primers	GenBank accession
***GSTA1.1***	663	F: 5'-ATGTCTGGGAAGCCAGTTCTG-3'	GQ228399
		R: 5'-TCAATGGAAAATTGCCATCA-3'	
***GSTA1.2***	666	F: 5′-ATGTCTGGGAAGCCAGTTCTG-3'	GQ228400
		R: 5′-TCAGTGGAAAATTGCTATCACACT-3'	
***GSTA1.3***	666	F: 5'-ATGTCTGGGAAGCCAGTTCT-3'	GQ228401
		R: 5'-TCAACTGAAAATTGCCAGCAG-3'	
***GSTA2***	669	F: 5'-ATGGCGGAGAAACCTAAGCTTCACTATACCA-3'	GQ228402
		R: 5'-TAATGTGAGGAAAATATTCAGTTTCTAAGGCCGC-3'	
***GSTA3***	672	F: 5'-ATGTCGGAGAAGCCCAGGCTCACCTA-3'	GQ228403
		R: 5'-TCAGTCTAGCTTAAAAATTTTCATCACAGTTGC-3'	
***GSTA4***	690	F: 5'-ATGGCTGCAAAACCTGTACTCTACTAC-3'	GQ228404
		R: 5'-CTAATTTGG TTTTACATCATAATACATCCGG-3'	

### Phylogenetic Analysis

Both nucleotide and amino acid sequences of the turkey GSTAs (EW, RGW, RP and DT), and chicken alpha class GSTs (cGSTAs) were aligned and compared using the phylogenetic and molecular evolutionary analysis software, MEGA version 5.05 (http://www.megasoftware.net/mega.php) [23]. Bootstrapped neighbor-joining method was used for phylogenetic reconstruction. A total of 500 bootstrap replicates were employed.

### Expression and Purification of Recombinant GSTAs in *E. coli*

Vector constructs for PCR were assembled using a strategy similar to that used in a previous study [21]for expression of six *GSTA* genes from domestic turkey (DT), which are also used here for comparison purposes. Sequencing revealed numerous allelic redundancies, so the following unique expression vectors were constructed **pGSTA1.1**: EW ( = RP), DT ( = RGW), **pGSTA1.2**: EW, RGW, RP, DT, **pGSTA1.3**: RGW ( = EW = RP), DT, **pGSTA2**: RGW, RP ( = EW), DT, **pGSTA3**: RP ( = EW), DT ( = RGW), pGSTA4: EW, RGW, DT ( = RP). To express His-tag fusion GSTA protein in *E. coli*, gene-specific primers with two restriction sites, *Nde*I at 5′-end (*BamH*I at 5′-end for *tGSTA4*) and *Xho*I at 3′-end, were designed to amplify the open reading frame (ORF) fused c-terminal 6X His tag to generate recombinant GSTA proteins using proofreading DNA polymerase (Table 2). The PCR profile was: 2 min at 94°C, 30 s at 94°C, 30 s at 60–63°C (25 cycles) and 1 min and 40 s at 72°C, followed by a final extension for 8 min at 72°C. Gel-purified gene PCR fragments were cloned into bacterial expression vector pET21α (Novagen, San Diego, CA), and then were transformed into *E. coli* strain BL21 (DE3). Colonies were inoculated to 30 mL of LB media containing ampicillin (100 mg/L) and grown overnight at 37°C. The starter culture was then inoculated (1∶100 dilution) into 500 mL of LB containing ampicillin (100 mg/L), and incubated at 37°C until OD_600_ reached 0.6–0.65 with shaking (250 rpm). After 3 h induction with 0.4 mM isopropyl-β-thiogalactopyranoside (IPTG), *E. coli* cells with the recombinant GSTA proteins were harvested and pelleted by centrifugation (5,000 g) at 4°C. Purification was by a modification of the manufacturer’s (QIAgen, Valencia, CA) instructions as follow: Harvested cell pellets were resuspended in lysis buffer (2.5 mM NaH_2_PO_4_, 300 mM NaCl, 10 mM imidazole, pH 8.0), disrupted by sonication (Fisher 60 Sonic, Fischer Scientific, Pittsburgh, PA) and lysates were centrifuged at 10,000 g for 50 min at 4°C. The clear supernatant was saved for purification. NI-NTA agarose was added to the supernatant and mixed by orbital shaker at 200 rpm for 60 min at 4°C. This mixture was loaded to the polypropylene column and the resins were washed twice by washing buffer (50 mM NaH_2_PO_4_, 2 M NaCl, 10 mM imidazole, pH 8.0, 50% glycerol, 1% Tween 20) and were eluted four times with elution buffer (50 mM NaH_2_PO_4_, 2 M NaCl, 250 mM imidazole, pH 8.0, 50% glycerol, 1% Tween 20). All eluates were pooled and used for SDS-PAGE, immunoblots and enzyme assays. Protein was then measured using the Bradford Protein Assay Kit (Bio-Rad, Hercules, CA).

**Table 2:** Primers with restriction sites for cloning and the 6X His-recombinant constructs of alpha-class *GSTAs* from wild and heritage turkey cDNA.

Gene	Construct	Primer sequences
***GSTA1.1***	pGSTA1.1	F: 5'-ATAGTCGCTCATATGTCTGGGAAGCCAGTTCTG-3'
		R: 5'-AAGCGAGGCTCGAGATGGAAAATTGCCATCAGACTT-3'
***GSTA1.2***	pGSTA1.2	F: 5'-TAGTCGCTCATATGTCTGGGAAGCCAGTTCT-3'
		R: 5'-ACGGAGGCTCGAGGTGGAAAATTGCTATCAC-3'
***GSTA1.3***	pGSTA1.3	F: 5'-GTAGTCGCTCATATGTCTGGGAAGCCAGTTCT-3'
		R: 5'-AGACCGGCTCGAGACTGAAAATTGCCAGCA-3'
***GSTA2***	pGSTA2	F: 5'-TTGCCGACATATGGCGGAGAAACCTAAGCTTCAC-3'
		R: 5'-CGACCGGCTCGAGGAAACTGAATATTTTCCTCAC-3'
***GSTA3***	pGSTA3	F: 5'-TATCATACATATGTCGGAGAAGCCCAGGCTCACC-3'
		R: 5'-CGACCGGCTCGAGGTCTAGCTTAAAAATTTTCATCAC-3'
***GST4***	pGSTA4	F: 5'-CTACGAGGATCCATGGCTGCAAAACCTGTACTCTACT-3'
		R: 5'-GGCCGCCTCGAGATTTGGTTTTACATCATAATACATCC-3'

### SDS-PAGE and Immunobloting

Purified recombinant tGSTA proteins were visualized by Coomassie Brilliant Blue staining on a 15% Tris-HCl Ready Gel (Bio-Rad, Hercules, CA). The SDS-PAGE gel was blotted onto PVDF membrane and blocked with 2% nonfat milk powder in TBST buffer (20 mM Tris-HCl, pH 7.6, 137 mM NaCl and 0.1% Tween 20) for 1 h at room temperature. After washing, the membrane was incubated with anti-His probe (1∶200) overnight at 4°C. On the next day, the membrane was incubated with bovine anti-mouse IgG HRP-conjugated secondary antibody (1∶8,000) for 1 h at room temperature and washed 5 times with TBST buffer for 60 min. Images were visualized by chemiluminescence using the Luminol reagent system and Cruz Marker TM molecular weight standard (Santa Cruz Inc, Santa Cruz, CA). Immunoblots were archived using a Nucleovision E20 Imaging Workstation (Nucleotech, Hayward, CA).

### Secondary Structures of Alpha-class GSTs

GenBank accession numbers of GSTA proteins (A1.1, A1.2, A1.3, A2, A3, A4) were assigned as follows: Eastern Wild (AET31416, AET31413, AET31410, AET31407, AET31404, AET31401), Rio Grande Wild (AET31417, AET31414, AET31411, AET31408, AET31405, AET31402), Royal Palm (AET31418, AET31415, AET31412, AET31409, AET31406, AET31403). Putative amino acid sequences of all GSTAs were aligned using ClustalW 2.0.12 (http://www.ebi.ac.uk/Tools/clustalw2/). Predictive secondary structures in wild and heritage turkey were compared with alpha-class GSTs of domestic turkey [20], [21] using PSIPRED Protein Structure Prediction Server software program (http://bioinf.cs.ucl.ac.uk/psipred/).

### Preparation of Hepatic Cytosols

Activities of recombinant turkey GSTAs were compared to those of their respective hepatic cytosols. Turkey liver cytosols were prepared as previously described [2]. All steps were at 4°C. Frozen liver was homogenized using a Polytron (Brinkman, Westbury, NY) in 2 vol of cold lysis buffer (50 mM Tris, 1 mM EDTA, 0.25 M sucrose, 150 mM KCl, 20 mM BHT and 200 mM PMSF buffer, pH 7.4). The homogenate was centrifuged at 600 g and then at 10,000 g for 10 min each. The supernatant was centrifuged at 16,000 g for 10 min and then was further centrifuged at 105,000 g for 1 h. The final supernatant (cytosols) was collected and stored at −80°C until use. As a comparison, liver cytosolic fraction from butylated hydroxyanisole (BHA)-induced Swiss Webster mice, the GSTs from which are reported to have the highest known conjugation activity due to the presence of mGSTA3 with high affinity toward AFBO [13] were included as a positive control, by methods we have published [21].

### Prototype GST Enzymatic Activities

Specific enzyme activities of *E. coli*-expressed GSTAs proteins and hepatic cytosolic GSTs were assayed for their conjugation activities toward substrates 1-chloro-2,4-dinitrobenzene (CDNB), 1,2-dichloro-4-nitrobenzene (DCNB), ethacrynic acid (ECA) [24], [25] and cumene hydroperoxide (CHP) [26], [27], which are used as prototype substrates to probe GST activities in various tissues. Because of ORF sequence redundancies, enzyme activities were determined only from those heterologously expressed proteins with unique sequences: GSTA1.1 (EW), GSTA1.2 (EW, RGW, RP), GSTA1.3 (RGW), GSTA2 (RGW, RP), GSTA3 (RP) and GSTA4 (EW, RGW). Heterologously-expressed GSTAs cloned from domestic turkey (DT) [21]were included as controls in all enzyme assays. Conditions of all assays were optimized in a final volume 1 mL containing 100 mM potassium phosphate buffer at room temperature (25°C) using spectrophotometer (Thermo Scientific, Madison, WI). Substrate and GSH concentrations optimized for each assay (run for 2 min) were: (a) 1 mM CDNB, 1 mM GSH, ΔA340 nm (Extinction coefficient: 9.6 mM^−1^ cm^−1^), buffer pH6.5 (b) 1 mM DCNB, 5 mM GSH, ΔA345 nm (8.5 mM^−1^ cm^−1^), buffer pH 7.5 (c) 0.2 mM ECA, 0.25 mM GSH, ΔA270 nm (5 mM^−1^ cm^−1^), buffer pH 6.5, and (d) glutathione peroxidase activity with (CHP) as substrate was determined with 1.2 mM CHP, 2 mM GSH, 1 U glutathione reductase, 0.2 mM NADPH, ΔA340 nm (6.22 mM^−1^ cm^−1^) and buffer pH 7.0.

### Determination of GST-mediated AFBO Conjugation Activity

Turkey liver microsomes and cytosolic fractions were prepared as previously described [14]. Glutathione S-transferase-mediated AFBO conjugation activity was measured for turkey hepatic GSTs [2], [16], and for *E. coli*-expressed GSTAs [21]. Turkey liver microsome (∼ 400 µg total protein) used to generate AFBO, were reacted with 100 µM AFB_1_ in spectral grade dimethyl sulfoxide, 2 mM NADPH, 5 mM GSH, and turkey cytosol (∼ 800 µg total protein) containing GST [2], [21], [28] or *E. coli-*expressed GSTAs (∼ 2 µg total protein). This mixture was incubated in AFBO trapping buffer (5 mM MgCl_2_, 25 mM KCl, 0.25 mM sucrose and 80 mM potassium phosphate, pH 7.6) to give a final volume of 250 µL, incubated at 37°C for 20 min with gentle shaking and stopped by adding 250 µL of cold MeOH spiked with 24 µM aflatoxin G_1_ (AFG_1_) as an internal HPLC standard. The samples were stored overnight at −20°C to facilitate protein precipitation and then centrifuged at 13,000 g for 10 min. The supernatants were filtered through a 0.2 µM nylon membrane (Millipore, Billerica, MA) and 100 µL was injected into the HPLC. Metabolites were separated on a Shimadzu LC system (Shimadzu, Pleasanton, CA), equipped with a model LC-20AD pump, a model SPD-20AV UV/vis detector, and an Econosphere C18 (150 mm×4.6 mm) column (Grace Davison Discovery Sciences, Deerfield, IL) kept at 4°C. The elution of the peaks was monitored by UV absorbance (λ = 365 nm). Mobile phases were used: solvent A was H_2_O adjusted to pH 3.65 with phosphoric acid and solvent B with 5% tetrahydrofuran in methanol. Amounts of metabolite formation were calculated by establishing calibration curves between the peak areas in the chromatograms and the amount of metabolite injected, using an authentic *exo*-AFB_1_-GSH standard.

### Statistical Analysis

Enzyme activities of hepatic cytosolic and recombinant GSTAs were compared and analysed by ANOVA and post hoc LSD for mean separation using SAS/STAT software with a level of significance set at *P*<0.05.

## Results

### Single Nucleotide Polymorphisms (SNPs)

The full-length cDNAs of six alpha-class GST genes (*GSTAs*) were isolated and cloned from the liver of two wild turkey subspecies (Eastern Wild: EW and Rio Grande Wild: RGW) and one heritage turkey breed (Royal Palm: RP). Each *GSTA* was identical in length to the corresponding homologs from domestic turkey (DT) (*GSTA1*.*1*, 663 bp; *GSTA1.2*, 666 bp; *GSTA1.3*, 666 bp; *GSTA2*, 669 bp; *GSTA3*, 672 bp; *GST4*, 690 bp) (Figures S1, S2, S3, S4, S5, S6). Nomenclature for wild and heritage turkey cytosolic GSTs were based on identities of the primary gene structures to the domestic homologs, and were assigned the following GenBank accessions: Eastern wild (*M. g. silvestris*), EWtGSTA1.1–EWtGSTA4: JN575076–JN575081; Rio Grande wild (*M.g.intermedia*), RGWtGSTA1.1–RGWtGSTA4: JN575082–JN575087; Royal Palm heritage breed, RPtGSTA1.1–RPtGSTA4: JN575088–JN575093.

**Figure 1:**
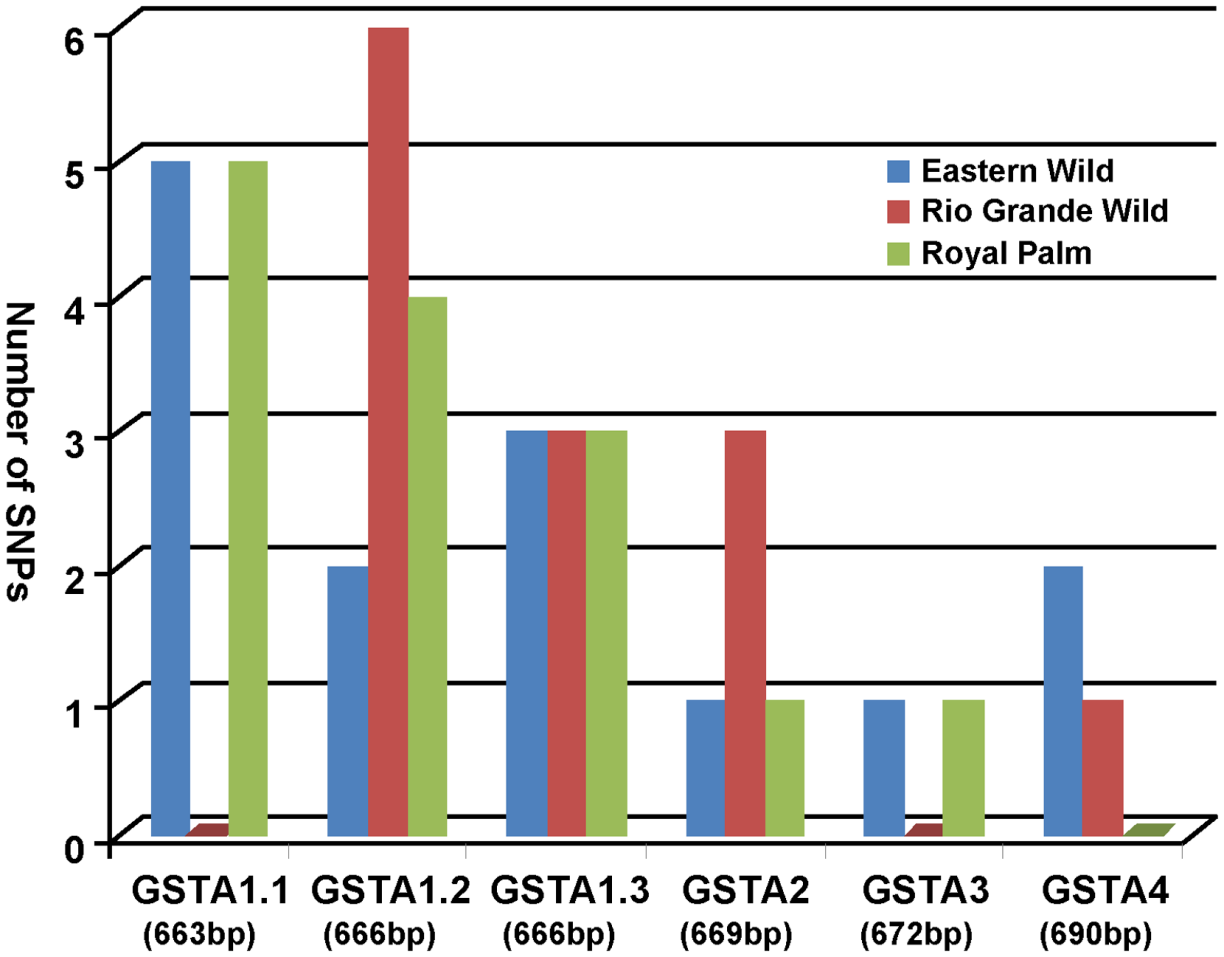
Genetic diversity of six Alpha class *GST* genes in four subspecies of turkeys. Domestic turkey was used for the reference to compare three subspecies. The Figure 1 shows the number of SNPs between domestic turkey and wild/heritage turkey. **DT**: domestic turkey, **EW**: Eastern Wild, **RGW**: Rio Grande Wild, **RP**: Royal Palm. Parenthesis (): the size of alpha class GST genes. The alpha class GST gene in subspecies showed the same nucleic acid sequences: ***tGSTA1.1***: DT = RGW, EW = RP; ***tGSTA1.3***: EW = RGW = RP; ***tGSTA2***: EW = RP; ***tGSTA3***: DT = RGW, EW = RP; ***tGSTA4***: DT = RP. *tGSTA1.2* showed the most diversity.

Sequencing revealed numerous putative SNPs within the protein coding region of *GSTAs* among the turkeys examined. As can be graphically seen in Figure 1, the allelic diversity as measured by frequency of SNPs in the turkeys investigated here - EW, RGW and RP - differed among the six *GSTA*s with respect to modern domestic turkeys (DT). The greatest allelic diversity in GSTA occurred in *GSTA1.1* and *GSTA1.2*, in which EW and RP turkeys contained 5 SNPs each, and 2, 6 and 4 SNPs in *GSTA1.2* for EW, RGW and RP, respectively. Sequences of *GSTA1.1* and *GSTA3* of RGW were identical to that of DT. The *GSTA1.3* variant in EW, RGW and RP possessed 3 SNPs compared to DT. There was only 1 SNP in *GSTA3* from EW and RP turkeys, and 2 and 1 SNP in *GSTA4* from EW and RGW, respectively, indicating that these genes possessed generally lower allelic variation. The identities and positions of mutations and predicted amino acids resulting from all SNPs are shown in Table 3. Four of the GSTA isoforms possessed the following encoding point mutations: GSTA1.1: Ser/Thr^12^, Ser/Phe^49^ and Thr/Ile^190^; GSTA1.2: Cys/Ser^49^, Leu/Phe^140^ and Gly/Trp^166^; GSTA1.3: Pro/Ser^172^ and Ser/Ala^125^ in GSTA3. The SNPs of *GSTA2* and *GSTA4* encoded silent mutations. Although one hallmark of adaptive evolution is a greater rate of substitution at non-synomymous than at synonymous sites, [29], we found that these mutations did not result in large differences of enzyme activities among GSTA isomers.

**Table 3:** Synonymous and non-synonymous SNPs in GSTM genes and point mutations in wild, heritage, and domestic turkeys.^1.^

Group	No SNPs	Enzyme	Nucleic acid position (bp)	Amino acid position (aa)^2^
			34 72 146 327 569	**12** 24 **49** 109 **190**
***GSTA1.1***		**tGSTA1.1**	T A C C C	Ser Ala Ser Asn Thr
(663 bp)	**5**	**EWtGSTA1.1**	A G T T T	Thr Ala Phe Asn Ile
		**RGWtGSTA1.1**	T A C C C	Ser Ala Ser Asn Thr
		**RPtGSTA1.1**	A G T T T	Thr Ala Phe Asn Ile
***GSTA1.2***			146 243 402 420 432 496 606	**49** 81 134 **140** 133 **166** 202
(666 bP)		**tGSTA1.2**	G C A G G G C	Cys Leu Pro Leu Gly Gly Ser
	**7**	**EWtGSTA1.2**	G T A G G G T	Cys Leu Pro Leu Gly Gly Ser
		**RGWtGSTA1.2**	C T G C A T C	Ser Leu Pro Phe Gly Trp Ser
		**RPtGSTA1.2**	G T A G A T T	Cys Leu Pro Leu Gly Trp Ser
***GSTA1.3***			487 514 558	163 **172** 186
(666 bp)		**tGSTA1.3**	T C C	Thr Pro Ala
	**3**	**EWtGSTA1.3**	C T A	Thr Ser Ala
		**RGWtGSTA1.3**	C T A	Thr Ser Ala
		**RPtGSTA1.3**	C T A	Thr Ser Ala
***GSTA2***			204 255 636	68 85 212
(669 bp)		**tGSTA2**	T T G	Thr Asp Ser
	**3**	**EWtGSTA2**	T T A	Thr Asp Ser
		**RGWtGSTA2**	C C A	Thr Asp Ser
		**RPtGSTA2**	T T A	Thr Asp Ser
***GSTA3***			373	**125**
(672 bp)		**tGSTA3**	T	Ser
	**1**	**EWtGSTA3**	G	Ala
		**RGWtGSTA3**	T	Ser
		**RPtGSTA3**	G	Ala
***GSTA4***			219 507	73 169
(690 bp)	**2**	**tGSTA4**	C A	Asn Glu
		**EWtGSTA4**	T G	Asn Glu
		**RGWtGSTA4**	C G	Asn Glu
		**RPtGSTA4**	C A	Asn Glu

1 Abbreviations are: t = domestic turkey; EW = Eastern Wild; RGW = Rio Grande Wild; RP = Royal Palm standard, or “heritage” turkey.

2 Amino acid positions of point mutations are in bold font.

### GST Phylogeny

**Figure 2:**
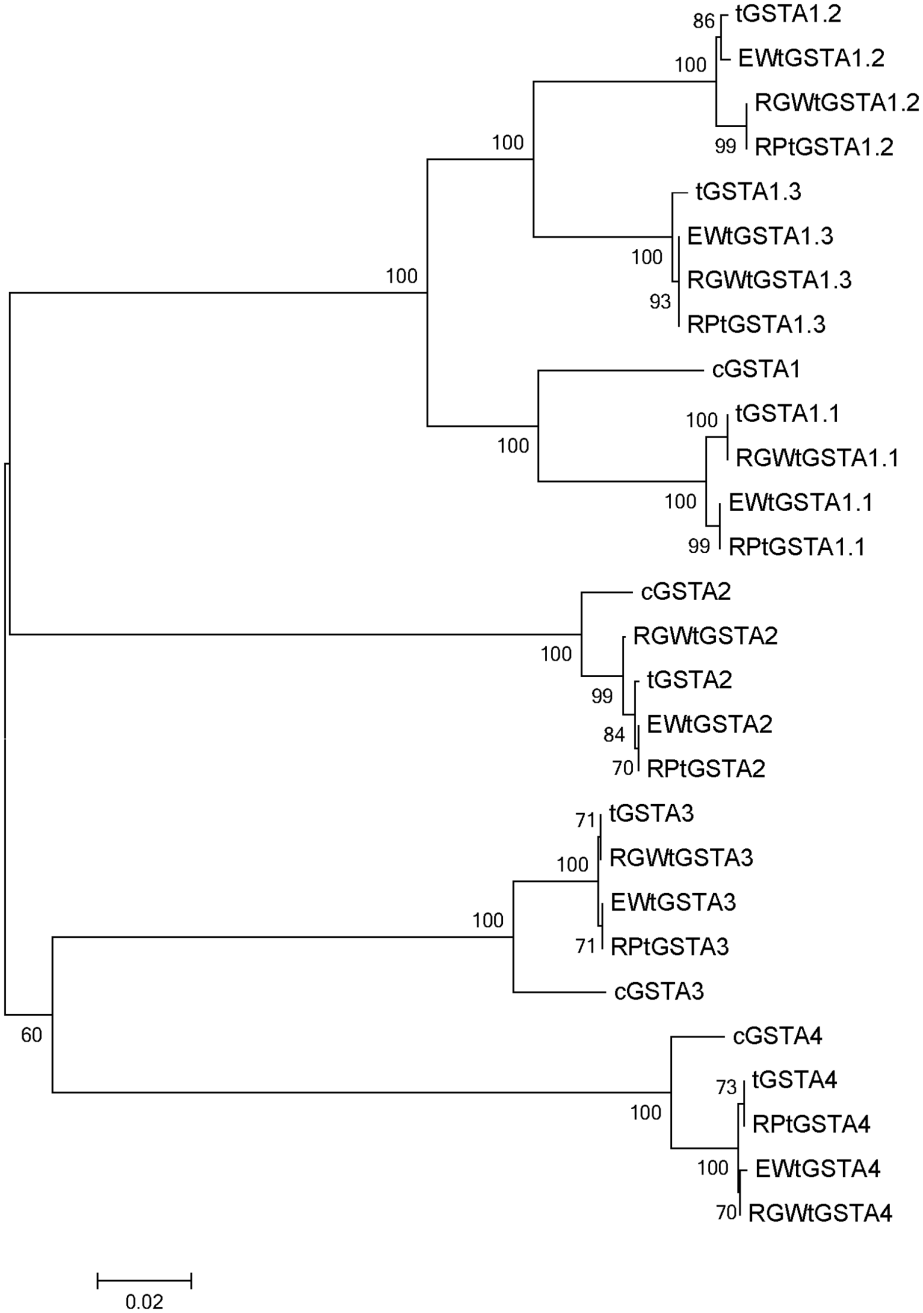
Phylogenetic analysis of amino acid sequences of turkey GSTAs along with chicken GSTAs. Genetic distance of *GSTAs* among wild (EWtGST: Eastern Wild, RGWtGST: Rio Grande Wild),heritage (RPtGST:Royal Palm) and domestic turkeys (tGST) and chicken alpha-class GST (cGSTA) determined using the neighbor-joining method. Bootstrap supporting values are indicated at each node. The scale bar represents phylogenetic distance (substitution/site). Identical results were obtained when nucleotide sequences were used for alignment and phylogenetic analysis.

Phylogenetic trees of the turkey GSTAs sequenced in this study (EW, RGW, heritage RP and domestic turkey DT) and those of chicken were constructed to illustrate the relatedness of the amplified GSTAs among these avian taxa (Figure 2). Clustering of nucleotide and amino acid sequences resulted in trees with identical overall topologies. In all cases the GSTA sequences of wild and heritage birds clustered with their domestic turkey homologs, confirming amplification of the target loci. On average, amino acid sequences of GSTAs from turkeys and chicken orthologues were 95% similar with the exception of chicken GSTA1 (cGSTA1) and domestic turkey tGSTA1.2 (81%), tGSTA1.3 (82%) [20], [21]. Clustering of the turkey and chicken sequences reflects the duplication of *GSTA*s prior to divergence of the two avian species.

### Secondary Structure of Alpha-class GSTs

**Figure 3:**
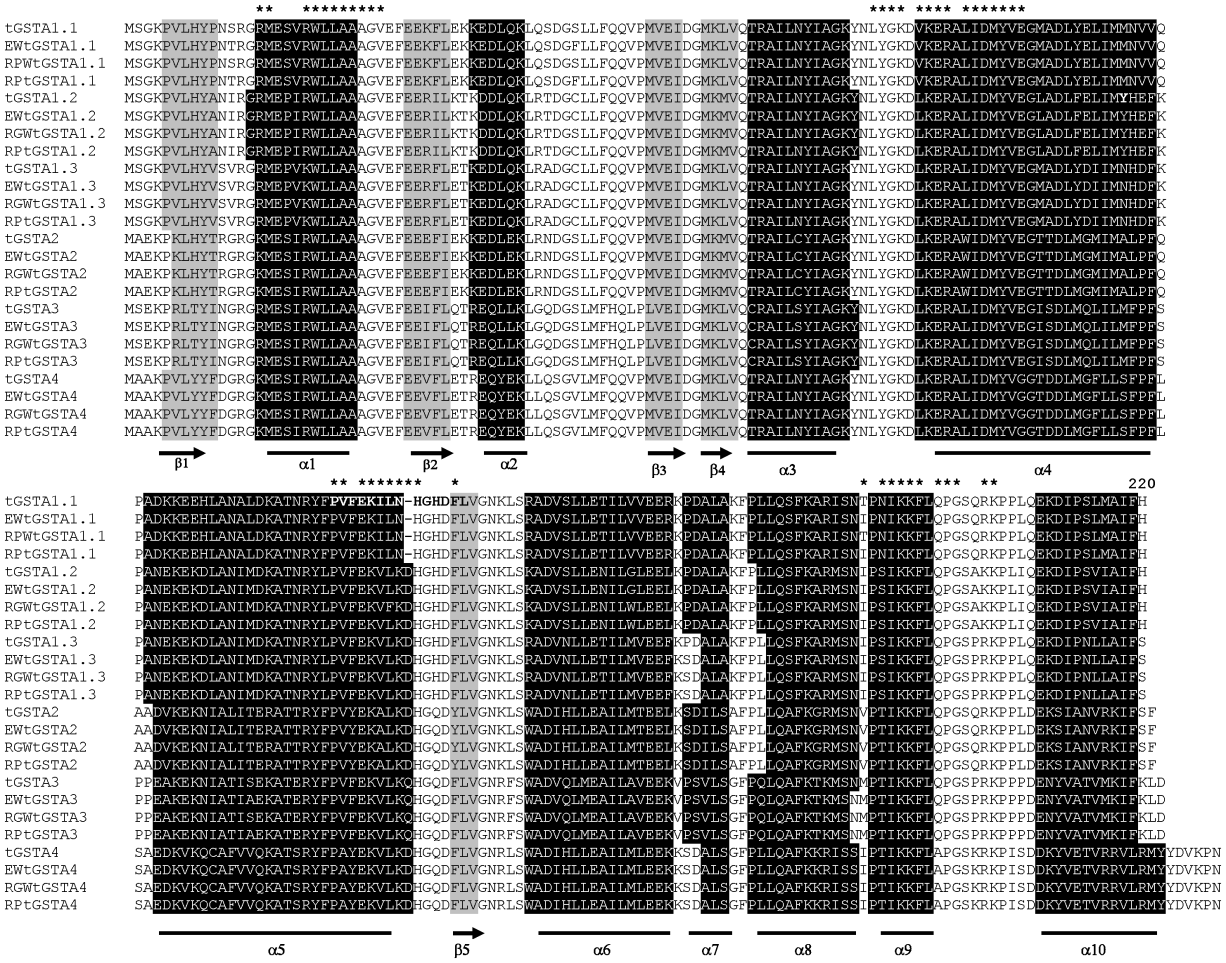
Multiple amino acid sequence alignments of six alpha-class GSTs from the wild, heritage and domestic turkeys with alpha-class GSTs. Underlines indicate alpha helices (black background) and arrows indicate beta strands (grey background). * indicates alpha-class signature motifs and specific conserved residues. The sequences of amino acid are (species name, GenBank accession no.): tGSTA1.1 (*M. gallopavo*, ACU44693), tGSTA1.2 (*M. gallopavo*, ACU44694), tGSTA1.3 (*M. gallopavo*, ACU44695), tGSTA2 (*M. gallopavo*, ACU44696), tGSTA3 (*M. gallopavo*, ACU44697), tGSTA4 (*M. gallopavo*, ACU44698), EWtGSTA1.1 (*M. g. silvestris*, AET31416), EWtGSTA1.2 (*M. g. silvestris*, AET31413JN575077), EWtGSTA1.3 (*M. g. silvestris*, AET31410), EWtGSTA2 (*M. g. silvestris*, AET31407), EWtGSTA3 (*M. g. silvestris*, AET31404), EWtGSTA4 (*M. g. silvestris*, AET31401), RGWtGSTA1.1 (*M. g. intermedia*, AET31417), RGWtGSTA1.2 (*M. g. intermedia,* AET31414), RGWtGSTA1.3 (*M. g. intermedia*, AET31411), RGWtGSTA2 (*M. g. intermedia*, AET31408), RGWtGSTA3 (*M. g. intermedia*, AET31405), RGWtGSA4 (*M. g. intermedia*, AET31402). RPtGSTA1.1 (*M. gallopavo*, AET31418), RPtGSTA1.2 (*M. gallopavo*, AET31415), RPtGSTA1.3 (*M. gallopavo*, AET31412), RPtGSTA2 (*M. gallopavo*, AET31409), RPtGSTA3 (*M. gallopavo*, AET31406), RPtGSTA4 (*M. gallopavo*, AET31403).

The amino acid sequences of wild and heritage turkey GSTAs were 98–100% similar to those of domestic turkeys. Like domestic turkeys, the open reading frame (ORF) of each gene was predicted to encode proteins in the range of 220–229 aa (25–26 kDa). Multiple sequence alignments comparing the amino acid residues as well as the secondary structure of GSTAs of the various turkey strains to those of revealed that all GSTAs contain a typical GST structure, consisting of two distinct conserved domains and four alpha-class signature motifs (Figure 3). Two domains consist of a thioredoxin-like N-terminal domain and a helical C-terminal domain. Like domestic breeds [20], all *GSTAs* from wild and heritage turkeys possess ten α-helices and five β-strands; N-terminal domain contains α1-β3/β1–4 and C-terminus contains α4-α10/β5. These alignments showed that all GSTAs possess four alpha-class signature motifs, except that GSTA1.1 lacked one motif (PVxEKVLKxHGxxxL) in residues 134–148 of the C-terminal domain, which did not appear to have a discernible effect on the catalytic activities of this form toward GST substrates (Table 4).

**Table 4:** Specific GST activity and GST-mediated detoxification of AFBO by unique recombinant Alpha-class GSTs and of hepatic cytosolic from wild, heritage and domestic turkeys.

	Specific enzyme activity (nmol/min/mg protein)					
	CDNB^1^	DCNB^2^	ECA^3^	CHP^4^	AFB_1_-GSH^5^	
**Recombinant GSTAs**						
tGSTA1.1*	1,674.61±48.15^a^	16.35±0.80^a^	44.31±2.00^ab^	451.83±12.41^ab^	19.54±1.42^abc^	
EWtGSTA1.1	850.45±13.08^bc^	16.02±0.58^a^	20.83±0.48^b^	1,977.13±35.11^c^	26.33±0.89^de^	
tGSTA1.2	7,220.94±75.81^d^	10.85±0.09^b^	164.51±6.18^c^	1,076.7±11.45^de^	22.49±1.97^be^	
EWtGSTA1.2	2,761.94±127.16^e^	5.70±0.16^c^	48.80±1.37^ad^	1,423.46±864.02^ef^	16.59±2.10^acf^	
RGWtGSTA1.2	3,137.45±234.93^f^	8.15±0.61^d^	69.92±0.96^d^	2,002.85±76.44^c^	16.62±2.02^acf^	
RPtGSTA1.2	3,161.92±15.18^f^	10.60±0.43^b^	95.40±7.36^e^	2,769.28±150.69^g^	21.97±2.54^be^	
tGSTA1.3	1,063.48±25.39^b^	1.46±0.15^e^	50.58±0.24^ad^	166.97±68.47^a^	21.98±2.26^be^	
RGWtGSTA1.3	638.89±12.60^cg^	1.40±0.05^e^	23.61±0.51^b^	1,924.87±486.10^cf^	29.10±1.50^d^	
tGSTA2	3,045.23±16.92^f^	7.55±0.59^d^	34.49±0.00^ab^	314.06±7.29^a^	21.71±0.54^be^	
RGWtGSTA2	1,880.01±61.97^a^	9.31±0.31^f^	29.58±0.69^ab^	287.88±7.91^a^	21.35±0.50^ab^	
RPtGSTA2	1,709.40±71.87^a^	7.23±0.24^d^	35.85±1.48^ab^	307.90±8.29^a^	19.42±0.94^abc^	
tGSTA3	4,254.39±37.32^h^	1.25±0.00^e^	174.32±1.54^c^	850.25±59.46^bd^	18.04±2.67^abf^	
RPtGSTA3	1,649.30±53.04^a^	1.23±0.21^e^	543.28±24.36^f^	1,934.90±30.79^cf^	21.42±1.43^b^	
tGSTA4	302.57±44.57^ij^	0.71±0.28^e^	21.38±0.98^b^	27.66±0.45^a^	20.06±0.33^abc^	
EWtGSTA4	504.92±91.97^gi^	0.86±0.27^e^	453.39±15.09^g^	90.99±3.49^a^	14.57±1.56^f^	
RGWtGSTA4	251.09±8.98^j^	1.06±0.42^e^	365.68±14.88^h^	91.39±3.52^a^	16.10±1.29^cf^	
**Hepatic GSTs**						
DT	1027.20±17.81^a^	1.51±0.05^a^	89.58±0.80^a^	180.87±0.97^a^	**n.d.**	
EW	714.09±37.10^b^	2.21±0.30^a^	90.52±3.75^a^	209.27±13.56^a^	0.018±0.004^a^	
RGW	524.14±14.53^c^	1.59±0.15^a^	81.06±1.98^a^	159.56±11.03^a^	0.028±0.007^a^	
RP	890.18±43.81^a^	2.61±0.09^a^	94.78±4.93^a^	203.91±8.50^a^	0.017±0.003^a^	
Mouse	2888.69±43.98^d^	64.55±1.10^b^	61.10±0.14^b^	805.54±43.68^b^	0.740±0.013^b^	

Under each column, different superscript letters represent significant difference (*P*<0.05). (A) Recombinant GSTs were analyzed by ANOVA and post hoc LSD was used for mean separation.

1 1-Chloro-2,4-dinitrobenzene: GSH (1 mM);

2 1,2-Dichloro-4-nitrobenzene: GSH (5 mM);

3 Ethacrynic acid; GSH (2.5 mM);

4 Cumene Hydroperoxide; GSH (2 mM);

5 AFB_1_-8,9-epoxide; GSH (5 mM); Mean ± SD of triplicate determination.

1 Abbreviations are: t or DT = domestic turkey; EW = Eastern Wild; RGW = Rio Grande Wild; RP = Royal Palm standard, or “heritage” turkey.

### Expression and Identification of Recombinant GSTA Proteins

**Figure 4:**
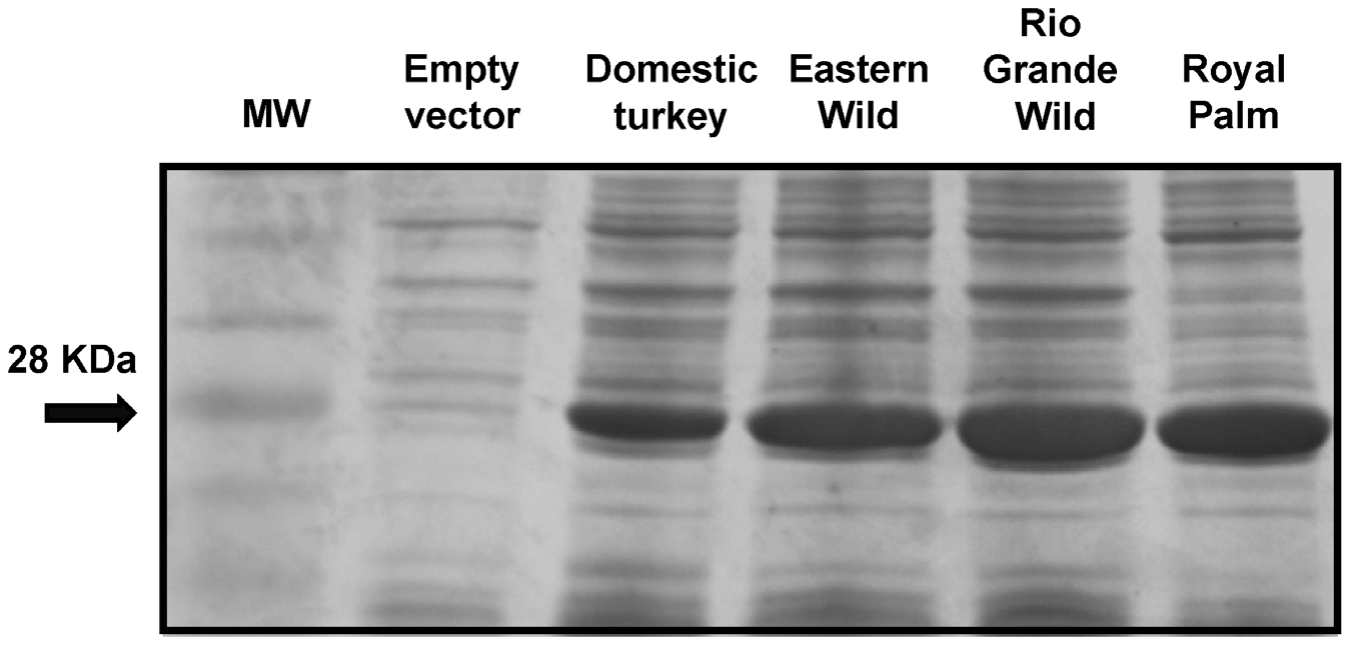
6X His-tagged purified alpha-class turkey GSTSA1.2 separated by SDS-PAGE gel (15%), visualized by Coomassie R-250 blue. Marker: molecular weight marker (MW); Lane 1, Empty vector (negative control); Lane 2, tGSTA1.2 (domestic); Lane 3, EWtGSTA1.2 (Eastern Wild); Lane 4, RGWtGSTA1.2 (Rio Grande Wild); Lane 5, RPtGSTA1.2 (Royal Palm heritage); Lane 6.

**Figure 5:**
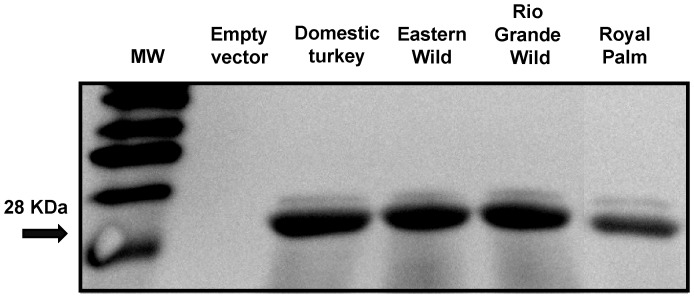
Western immunoblot showing expression of His-tag recombinant purified soluble alpha-class turkey GSTA1.2. Anti-his primary antibody and HRP-conjugated secondary antibody were used and bands were detected by chemiluminescence analysis. MW: molecular weight marker; Lane 1, Empty vector (negative control); Lane 2, tGSTA1.2 (domestic turkey); Lane 3, EWtGSTA1.2 (Eastern Wild); Lane 4, RGWtGSTA1.2 (Rio Grande Wild); Lane 5, RPtGSTA1.2 (Royal Palm heritage); Lane 6.

Recombinant 6X His-tagged clones in pET21α (**pGSTA1.1**: EW DT, **pGSTA1.2**: EW, RGW, RP, DT, **pGSTA1.3**: RGW, DT, **pGSTA2**: RGW, RP, DT, **pGSTA3**: RP, DT, pGSTA4: EW, RGW, DT) were expressed in *E. coli* BL21 (DE3). The molecular mass of 6X His-tagged recombinant GSTAs were predicted as follows: GSTA1.1∶26.3 kDa; GSTA1.2∶26.4 kDa; GSTA1.3∶26.5 kDa; GSTA2∶26.7 kDa; GSTA3∶26.7 kDa; and GSTA4∶28.8 kDa. A series of affinity-purified 6X His-tagged GSTA1.2 recombinant proteins from domestic, Eastern Wild, Rio Grande Wild and Royal Palm heritage separated on SDS/PAGE revealed the expected band with a MW of 28 kDa (Figure 4). Protein bands of the same mass were also observed in immunoblots (Figure 5). Other GSTAs had a similar appearance on SDS/PAGE and in immunoblots (data not shown).

### GST-specific Catalytic Activities of Recombinant GSTAs and Hepatic Cytosolic Fractions

Activities of cDNA-expressed recombinant GSTAs as well as their hepatic cytosolic forms toward conjugation of CDNB, DCNB, ECA and CHP were measured and compared under standard conditions (Table 4). All recombinant GSTA proteins possessed detectable enzymatic activities toward these prototype substrates, with few discernible trends where one recombinant protein consistently showed either high or low conjugation activity. However, the highest activity toward CDNB was observed for tGSTA1.2 cloned from domestic turkey and the GSTA3 allele cloned and expressed from RP had substantially higher ECA and CHP activity than all others. As we have previously observed in GSTA1.1 from domestic turkey [20], [21], all GSTA1.1 isoforms lack a signature motif (PVxEKVLKxHGxxxL; residues 134–148) in the C-terminal domain. However, this did not appear to discernibly affect enzyme activity of these recombinant, expressed isoforms. Indeed, the specific enzyme activities of GSTA1.1 were well within the range of that seen from other isoforms.

Activities toward such prototype substrates are important in confirming that expressed GSTs are catalytically active proteins. However, activities toward these substrates are not associated with that against specific substrates such as AFB_1_. When GST activity toward AFBO was measured all recombinant GSTAs possessed detoxification activity toward this *in situ* - generated reactive intermediate (Table 4). Recombinant GSTA1.1 and A1.3 from EW and RGW birds appeared to have the highest AFBO trapping activity. This is especially interesting given the absence of the motif in residues 134–148 in the former protein. Unlike recombinant and expressed GSTs, there were significant differences in the conjugation activities of hepatic cytosols. While liver cytosols from EW, RGW, and RP turkeys had substantial AFBO trapping activity, the DT cytosols did not.

Liver cytosol from BHA-induced mice, which expresses high amounts of mGSTA3, the “gold standard” of AFBO-conjugating activity, possessed AFBO-conjugating activity 41, 44 and 26-fold greater than that in livers from EW, RP and RGW, respectively. The rate of AFBO conjugation measured in mouse liver was close to that published for similarly BHA-induced Swiss Webster mice [30].

## Discussion

Susceptibility of animals and humans to the toxic and carcinogenic properties of AFB_1_ arises from a complex process involving a balance between P450-mediated bioactivation of AFBO, and the efficiency with which this reactive and toxic intermediate is detoxified, principally through GST conjugation [11]. Among animal species studied to date, turkeys are one of the most susceptible [2], [15], [21]. In turkey liver, AFB_1_ is rapidly metabolically activated to the putative toxic and carcinogenic intermediate AFBO, by high-efficiency cytochromes P450 1A5 [31] and P450 3A37 [6], though the former is responsible for >98% of bioactivation at pharmacological concentrations of AFB_1_ in the liver [6].Bioactivation by P450 s is a critical and requisite step for the toxicity of thousands of drugs and environmental chemicals. In the case of AFB_1_, it appears that the presence of hepatic GSTs with ability to catalytically conjugate GSH with AFBO is the principal “rate-limiting” determinant for AFB1 toxicity, regardless of the efficiency of AFB_1_ bioactivation [13]. Studies in our laboratory have consistently shown that while livers from domestic turkeys have GST activity against prototype substrates, they are deficient in expression of GSTs with affinity activity toward AFB_1_ [2], [15], [16]. Wild turkeys have been shown to be relatively AFB_1_-resistant [17], [32], and the fact that livers from wild turkeys, unlike their closely-related domestic counterparts, possess functional AFBO-trapping GSTs, supports the view that GSTs are critical to AFB_1_ susceptibility and resistance in animals. In this study, six alpha-class GST subunits from AFB_1_-resistant turkeys were cloned and expressed in *E. coli* in order to explore potential sequence differences that might affect catalytic activity of these proteins toward GST prototype substrates and toward enzymatically generated AFBO.

Domestication is a complex process, with human-animal interactions that vary considerably in terms of the degree of human intervention and the intensity of selection [33]. Genetic changes have accompanied animal domestication and breed development as domestic animals pass through severe bottlenecks during selection from wild counterparts for human benefit [34] Significant loss of species-wide minor alleles in commercial populations is well documented [19]. A number of genetic diversity studies in chicken have reported loss of genetic diversity in commercial populations because of high selection pressure and low effective population size [35], [36]. Genome-wide assessment of chicken SNP genetic diversity indicates significant absence of rare alleles (50% or more) in commercial breeds compared to ancestral breeds [19]. Whole genome SNP discovery in the turkey has identified 5.49 million putative SNPs, and comparative analyses indicate that specific haplotypes have been selected in the modern domesticated bird [37].

With a rich historic background [22], turkeys are the fourth-largest in US animal production [38], but little is known about the genetic diversities of wild ancestors compared to commercially domesticated avian species. Recent studies on the major histocompatibility complex (MHC) revealed increased haplotype variability in wild turkey as compared to domestic turkeys [39], [40]. The domestic turkey is recognized as a single breed with eight different varieties defined primarily by plumage color [41], [42]. Sub-specific classification of wild turkeys is supported by analysis of nuclear and mitochondrial DNA markers [43]. Phylogenetic comparison of the amino acid sequences of turkey and chicken GSTAs indicates that turkey GSTAs are orthologous to chicken GSTAs [20]. Although it has been reported that GSTA subfamilies arose from gene duplications that subsequently diverged in each species through evolution [32], our study confirmed that the *GSTA* loci in turkey and chicken were established prior to speciation.

A significant observation in this study was that hepatic cytosols from wild and heritage turkeys possessed significant catalytic detoxification capacity toward the carcinogenic intermediate AFBO, while such activity from those from domestic birds was not detectable. On the other hand, *E. coli*-expressed GSTAs expressed from both domestic, wild and heritage breeds all possessed AFBO conjugating activity. There are many possible explanations for this observation. Down-regulation of GSTs is known to occur through a variety of mechanisms, and is often associated with variations in enzyme activity. In humans, two non-synonymous SNPs in the electrophile-binding region of GST Pi 1 (*GSTP1*) isolated from malignant gliomas appear to be associated with reduced GSTP1-related enzyme activity compared to normal glioma tissue [44]. Variants of GST M1, T1 and P1 were associated with reduced oxyplatin-induced neuropathy in cancer patients predictive of differences in enzyme activities related to these genes [45].

Numerous functional, allelic SNPs are known to exist within regulatory elements of human GSTAs resulting in attenuation or loss of enzyme activity [46]. Epigenetic regulation of *GSTA* gene expression or post-translational modifications may also be in play, suppressing GSTAs enzymatic activities in the livers of domestic turkeys. It is possible that artificial selection has reduced polymorphism levels [43] with significant allelic loss of hepatic GSTs in domestic commercial turkeys resulting from selective pressures on genes related to economically-desirable traits such as higher growth rates and meat production [33].

A recent study on the inheritance of epigenetic variation demonstrated a difference in the profile of brain gene expression and promoter methylation between domesticated White Leghorn layers and their wild ancestors, the Red Jungle fowl [45] speculated to be a result of either selection of genotypes affecting epigenetic states, or inheritable methylation states during chicken domestication. The mechanism of genetic and epigenetic variation and inheritance of hepatic *GSTA* gene between wild and domestic turkeys is the subject of current studies in our laboratory.

The catalytic activity of turkey GSTAs toward AFBO were substantially lower than that for mGSTA3, the “gold standard” detoxification activity used here as positive control. However, there is evidence that low expression of GSTs is functionally important in animals. For example, human hepatocytes with one or two functional GSTM1 alleles had three-fold lower lower AFB_1_-DNA adducts than GSTM1 null hepatocytes [47]. Thus, it seems reasonable that the relatively low specific activity of turkey GSTAs may well account for the observed phenotypic differences in susceptibility between wild and domestic turkeys.

In this study, neither of the synonymous mutations in *GSTA2* or *GSTA4*, nor non-synonymous mutations in *A1.1*, *1.2*, *1.3* and *A3* appeared to result in measurable differences in enzyme activities of the *E. coli*-expressed enzymes. This observation implies that regulation of GSTAs in turkey liver also occur in regions outside the coding sequence. There is ample evidence for this in humans where hypermethylation in the promoter region of *GSTP1* results in reduced *GSTP1* expression affecting hepatocellular, breast, renal, lung, and colon cancer, as well as some lymphomas [35], [48], [49].

One goal of this research was to determine the extent to which wild and heritage turkeys might contain potentially useful GST alleles not found in domestic birds. As a result of the difficulties in improving AFB_1_ resistance in domestic turkey by traditional selection, the achievement of such improvement is one of the most important applications of genome research [34], [50]. We are currently investigating the transcriptome of birds following AFB_1_ exposure and further examining the interactions of the proteins of alpha-class and other GSTs.

## Supporting Information

Figure S1 **Alignment of nucleotide sequences and positions of five single nucleotide polymorphisms (SNPs) in the coding region of hepatic α-class GSTA1.1 amplified from domestic (tGSTA), Rio Grande Wild (RGWtGST), Eastern Wild (EWtGST), and Royal Palm heritage (RPtGST) turkeys (663 bp; Genbank accessions GQ228399, JN575082, JN575076, JN575088, respectively).** Periods denote identical bases for alignment.
(TIFF)

Figure S2 **Alignment of nucleotide sequences and positions of seven single nucleotide polymorphisms (SNPs) in the coding region of hepatic α-class GSTA1.2 amplified from domestic (tGSTA), Rio Grande Wild (RGWtGST), Eastern Wild (EWtGST), and Royal Palm heritage (RPtGST) turkeys (666 bp; Genbank accessions GQ228400, JN575083, JN575077, JN575089, respectively).** Periods denote identical bases for alignment.
(TIFF)

Figure S3 **Alignment of nucleotide sequences and positions of three single nucleotide polymorphisms (SNPs) in the coding region of hepatic α-class GSTA1.3 amplified from domestic (tGSTA), Rio Grande Wild (RGWtGST), Eastern Wild (EWtGST), and Royal Palm heritage (RPtGST) turkeys (666 bp; Genbank accessions GQ228401, JN575084, JN575078, JN575090, respectively).** Periods denote identical bases for alignment.
(TIFF)

Figure S4 **Alignment of nucleotide sequences and positions of three single nucleotide polymorphisms (SNPs) in the coding region of hepatic α-class GSTA2 amplified from domestic (tGSTA), Rio Grande Wild (RGWtGST), Eastern Wild (EWtGST), and Royal Palm heritage (RPtGST) turkeys (669 bp; Genbank accessions GQ228402, JN575085, JN575079, JN575091, respectively).** Periods denote identical bases for alignment.
(TIFF)

Figure S5 **Alignment of nucleotide sequences and positions of one single nucleotide polymorphism (SNP) in the coding region of hepatic α-class GSTA3 amplified from domestic (tGSTA), Rio Grande Wild (RGWtGST), Eastern Wild (EWtGST), and Royal Palm heritage (RPtGST) turkeys (672 bp; Genbank accessions GQ228403, JN575086, JN575080, JN575092, respectively).** Periods denote identical bases for alignment.
(TIFF)

Figure S6 **Alignment of nucleotide sequences and positions of two single nucleotide polymorphism (SNP) in the coding region of hepatic α-class GSTA4 amplified from domestic (tGSTA), Rio Grande Wild (RGWtGST), Eastern Wild (EWtGST), and Royal Palm heritage (RPtGST) turkeys (690 bp; Genbank accessions GQ2284042, JN575087, JN575081, JN575093, respectively).** Periods denote identical bases for alignment.
(TIFF)
